# A Handy Book on Opthalmic Surgery, for the Use of Practitioners

**Published:** 1867-02

**Authors:** 


*A Handy Book on Opthalmic Surgery, for the use of Practitioners.* By John Z. Lawrence, F. R. C. S., M. B. University of London, Surgeon to the Opthalmic Hospital, Southwark; Editor of the Opthalmic Review, &c.; and Robert C. Moon, House Surgeon to the Opthalmic Hospital, Southwark, with numerous illustrations.

This is a valuable book, and should be in the hands of every practitioner.

